# Impact of T Cell Ratios on Survival in Pleural Mesothelioma: Insights from Tumor Microenvironment Analysis

**DOI:** 10.3390/cancers16193418

**Published:** 2024-10-08

**Authors:** Laura V. Klotz, Andreas Weigert, Florian Eichhorn, Michael Allgäuer, Thomas Muley, Rajiv Shah, Rajkumar Savai, Martin E. Eichhorn, Hauke Winter

**Affiliations:** 1 Department of Thoracic Surgery, Thoraxklinik, University Hospital Heidelberg, Röntgenstraße 1, 69126 Heidelberg, Germany; florian.eichhorn@med.uni-heidelberg.de (F.E.); martin.eichhorn@med.uni-heidelberg.de (M.E.E.); hauke.winter@med.uni-heidelberg.de (H.W.); 2 Translational Lung Research Center Heidelberg (TLRC), German Center for Lung Research (DZL), Im Neuenheimer Feld 156, 69120 Heidelberg, Germany; 3 Institute of Biochemistry I, Faculty of Medicine, Goethe-University Frankfurt, Theodor-Stern-Kai 7, 60590 Frankfurt, Germany; weigert@biochem.uni-frankfurt.de; 4 Institute of Pathology, University Hospital Heidelberg, Im Neuenheimer Feld 224, 69120 Heidelberg, Germany; michael.allgaeuer@med.uni-heidelberg.de; 5 Translational Research Unit, Thoraxklinik, University Hospital Heidelberg, Röntgenstraße 1, 69126 Heidelberg, Germany; thomas.muley@med.uni-heidelberg.de; 6 Department of Thoracic Oncology, Thoraxklinik, Heidelberg University Hospital, Röntgenstraße 1, 69126 Heidelberg, Germany; rajiv.shah@med.uni-heidelberg.de; 7 Lung Microenvironmental Niche in Cancerogenesis, Institute for Lung Health, Justus Liebig University, 35392 Giessen, Germany; rajkumar.savai@mpi-bn.mpg.de

**Keywords:** pleural mesothelioma, t cell, tumor microenvironment, immune cell, PD-L1, epithelioid, sarcomatoid, thoracic cancer

## Abstract

**Simple Summary:**

Although immunotherapy is well established for treating pleural mesothelioma, it has not shown a breakthrough in improving the overall survival of these patients. A detailed study of the tumor microenvironment could lead to a better understanding of the factors that influence tumor growth to optimize treatment. In our cohort, T cell infiltrate differences were strongly associated with overall survival.

**Abstract:**

**Background:** Immunotherapy has significantly improved overall survival in patients with pleural mesothelioma, yet this benefit does not extend to those with the epithelioid subtype. Tumor growth is believed to be influenced by the immune response. This study aimed to analyze the tumor microenvironment to gain a better understanding of its influence on tumor growth. **Methods:** The tumor immune cell infiltration of 188 patients with pleural mesothelioma was characterized by multiplex immunofluorescence staining for CD3+ cells (CD3+), CD4+ cells (CD3+/CD4+), CD8+ cells (CD3+/CD8+), Treg (CD3+/CD4+/CD8-/CD163-/Foxp3+), PD1 cells (PD1+), and T helper cells (CD3+/CD4+/CD8-/CD163-/FoxP3-). The distribution of specific immune cells was correlated with clinical parameters. **Results:** A total of 188 patients with pleural mesothelioma (135 epithelioid, 9 sarcomatoid, 44 biphasic subtypes) were analyzed. The median age was 64.8 years. Overall survival was significantly longer in the epithelioid subtype than in the non-epithelioid subtype (*p* = 0.016). The presence of PD-L1 expression had a negative effect on overall survival (*p* = 0.041). A high ratio of CD4+ cells to regulatory T cells was associated with a significantly longer overall survival of more than 12 months (*p* = 0.015). The ratio of CD4+ cells to regulatory T cells retained its significant effect on overall survival in the multivariate analysis. **Conclusions:** Distinct differences in the T cell immune infiltrates in mesothelioma are strongly associated with overall survival. The tumor microenvironment could therefore serve as a source of prognostic biomarkers.

## 1. Introduction

Pleural mesothelioma (PM) arises from mesothelial cells of the parietal and visceral pleura. It is primarily associated with previous exposure to asbestos and occurs predominantly in male patients due to occupational risks. Due to the late onset of symptoms and delayed diagnosis, PM is often diagnosed at an advanced stage when the disease has already infiltrated the surrounding structures. The therapeutic options are further limited by advanced age and comorbidities [1]. Current therapeutic approaches for the treatment of PM are based on immune checkpoint inhibitors and conventional chemotherapy [2]. For patients with a non-epithelioid histology or advanced disease, immunotherapy with nivolumab and ipilimumab has become the treatment of choice [3]. In the large subgroup of epithelioid PM patients, immunotherapy has not been shown to a lead to a breakthrough improvement in overall survival (OS) compared to chemotherapy [3]. Nevertheless, immunotherapy has become the standard of care [2]. The response to immunotherapy varies greatly from patient to patient, and most patients have an unsatisfactory median overall survival of less than two years [2].

In the early stages of the disease, a multimodal treatment concept suitable for this highly selective group of patient is a promising strategy with a comparatively long overall survival [3,4]. Careful patient selection, particularly due to the recent discussion about the results of multimodal therapy, including surgery, in the MARS-2 study, is crucial to define the appropriate treatment approach for individual patients [5,6]. Clinical trials are currently being conducted to evaluate the additional benefit of immunotherapy in multimodal treatment approaches [7].

Several morphological features like histopathological subtype or nuclear grade are of prognostic value in PM. Currently, there is no reliable biomarker to predict disease progression and therapeutic response in patients with PM. Similar to NSCLC (Non-Small Cell Lung Cancer) and other solid malignancies, an exploratory analysis in the Checkmate-743 trial failed to show any predictive value of programmed cell death-ligand 1 (PD-L1) [3]. An improved understanding of the tumor microenvironment of PM is urgently needed for the identification of predictive biomarkers for immune checkpoint inhibitors [8]. A detailed analysis of the tumor tissue could help to identify relevant biomarkers that can predict the progression of the disease and the response to therapy. In connection with the immune response, it is assumed that lymphocytes and T cells in particular are heavily involved in the regulation of tumor growth [9]. 

The aim of this study was, therefore, to analyze the immune tumor microenvironment of different PM subtypes with regard to the prognostic influence of specific T cell characteristics on tumor growth.

## 2. Materials and Methods

### 2.1. Study Population

We retrospectively reviewed the clinical data of 188 patients who underwent surgery for a diagnosis of PM. From these patients, we retrospectively selected tissue sections obtained by surgical biopsy for histologic confirmation between 05/2001 and 03/2011 at the Department of Thoracic Surgery, Thoraxklinik Heidelberg. Patient characteristics and survival data were obtained from our institutional database. The tumor stage of all patients was classified according to the 8th edition of the TNM staging system [10]. The study was approved by the Institutional Review Board of the University of Heidelberg (No. S-174/2019).

### 2.2. Tissue Processing

To analyze the tumor microenvironment, tissue microarrays (TMAs) containing representative areas of tumor tissue from 188 mesothelioma patients were prepared according to the standard operating procedures of the Tissue Biobank of the National Center for Tumor Diseases (NCT, Heidelberg, Germany). As a quality criterion, areas that contained more than 80% of tumor cells without any necrosis were selected by the pathologist. TMAs were prepared as previously described: two to three cylindrical cores (1 mm diameter) of tumor tissue were removed from the donor block and transferred to a recipient block using an automated TMA robot (TMA Grand Master, 3D Histech, Budapest, Hungary) [8].

### 2.3. Multiplex Immunohistochemistry and Image Analysis 

Tissue sections were stained with Opal 7-Color Automation IHC Kits (Akoya Bioscience, Marlborough, MA, USA) in the BOND-RX Multiplex IHC Stainer (Leica, Wetzlar, Germany). Each tissue section was stained in 6 consecutive rounds, including blocking with 5% BSA and subsequent incubation with primary antibodies, corresponding secondary HRP-conjugated antibodies, and Opal fluorophores as described previously [11,12]. Nuclei were counterstained with 4′,6-diamidino-2-phenylindole (DAPI), which is included in Opal 7-Color Automation IHC kits, and slides were mounted with Fluoromount-G (Southern Biotech, Birmingham, AL, USA).

Imaging was performed using the PhenoImager HT imaging system (Akoya Bioscience) and images were analyzed using the phenotyping application from the inForm software V2.4.10 (Akoya Bioscience). Based on the DAPI staining, cell segmentation was performed using the implemented real-time algorithm for matched cell detection. Phenotyping of the different immune cell populations investigated was based on (simultaneous) positive staining for the biomarkers used (Table 1). The histopathological analysis focused on the identification, differentiation, and quantification of the T cell infiltrate, including its specific phenotypes. PD-L1 expression was stained separately on another slide in the TMA to further validate the staining (Klon: SP263; Ventana Benchmark Ultra, Tucson AZ, USA).

### 2.4. Statistical Analysis 

The total number of cells for each stained marker and cell phenotype was analyzed. The median of the different regions of interest per patient (two to three regions of interest per tumor sample, depending on the amount of tumor tissue available) was calculated. All values represent the cell counts for each marker per 10,000 T cells counted. The relationships between cell counts and patient characteristics were assessed and calculated using the Wilcoxon rank test and the Mann–Whitney U test, respectively. 

Data were collected and analyzed using SigmaPlot v14.0 (Systat Software Inc., Erkrath, Germany) and Prism v7.0 (GraphPad, San Diego, CA, USA). Overall survival was defined as the time from the date of first treatment to tumor-related death or last follow-up. The Kaplan–Meier method was used to perform the survival analysis. Differences in survival between groups were calculated using the log-rank test. Hazard ratios (HR) were estimated using Cox proportional hazards univariate and multivariate models. A *p*-value of less than 0.05 was considered statistically significant.

## 3. Results

### 3.1. Patient Characteristics

The study population consisted of 175 male and 13 female patients with histologically confirmed pleural mesothelioma. Of these, 135 (71.8%) had an epithelioid, 9 (4.8%) a sarcomatoid and 44 (34.4%) a biphasic subtype. The median age at diagnosis was 64.8 years. All patients were diagnosed by surgical biopsy at the Thoraxklinik Heidelberg. Approximately 65.4% of patients had known asbestos exposure. The median follow-up time was 15.1 months from date of diagnosis. The tissue samples used in this study were obtained during diagnostic thoracoscopy prior to initiation of therapy. All patients received platinum-based chemotherapy as an initial treatment. None of the patients were alive at the end of the follow-up period. More detailed information on patient characteristics is shown in Table 2.

### 3.2. Patient Outcome

**Figure 1:**
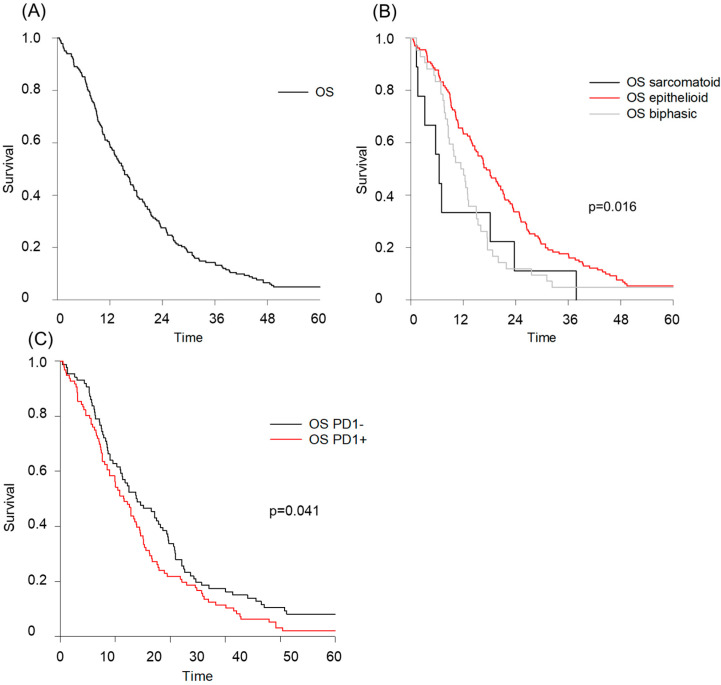
Overall survival data for pleural mesothelioma cohort stratified by histology and PD-L1 status. (**A**) Kaplan–Meier overall survival (OS) curves for the entire cohort (n = 188) with a median OS of 15.1 months. (**B**) OS for patients stratified by mesothelioma histology. Epithelioid subtype with an OS of 17.3 months, biphasic subtype with an OS of 11.4 months and sarcomatoid subtype with an OS of 6.4 months (*p* = 0.016). (**C**) OS for the entire cohort with distribution according to PD-L1 status. Patients without PD-L1 expression had a significantly better OS (*p* = 0.041).

The median overall survival (OS) for the entire cohort was 15.1 months with an interquartile range (IQR) of 8.3–25.4 months, as shown in Figure 1A. Among the tumor subtypes, the median OS differed significantly between the epithelioid, biphasic, and sarcomatoid tumor subtypes at 17.3, 11.4, and 6.4 months, respectively (*p* = 0.016; Figure 1B). The expression of PD-L1 in mesothelioma tissue was positive (defined as PD-L1 ≥ 1%) in 97 patients (51.6%). A total of 91 patients (34.4%) were classified as PD-L1-negative. Only 32 patients (17.0%) had a PD-L1 expression of >50% (Table 2). After stratifying patients according to their PD-L1 status, OS was significantly better in patients who did not express PD-L1 (*p* = 0.041; Figure 1C).

### 3.3. The Immune Cell Infiltrate

**Figure 2:**
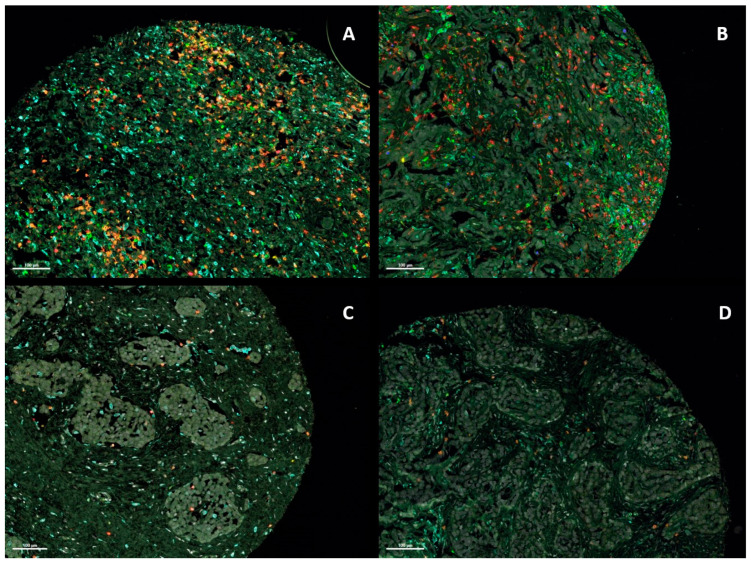
Representative multispectral images of pleural mesothelioma tumor tissue for the validation of prognostic T cell markers. The tissue was stained with the antibodies CD3 (red), CD8 (orange), CD4 (green), PD-1 (yellow), Foxp3 (blue), and CD163 (cyan). The cell nuclei were counterstained with DAPI (white). Scale bar: 100 μm. (**A**,**B**) Representative immunofluorescence staining of PM TMAs showing strong infiltration of immune cells into the mesothelioma tumor tissue. (**C**,**D**) Representative immunofluorescence staining of PM TMAs representing mesothelioma tumor tissue with low accumulation of immune cells.

The median CD4-positive (CD4+) cell count (per 10,000 T cells) was 294.51 for the entire cohort and 315.54 for the biphasic, 294.12 for the epithelioid, and 219.78 for the sarcomatoid subtype. The median CD8+ cell count (per 10,000 T cells) for all patients was 882.35. By histologic subtype, the median CD8-positive (CD8+) cell count was 807.77 for biphasic, 882.35 for epithelioid, and 1208.79 for sarcomatoid subtype. The median number of regulatory T cells (Treg) per 10,000 T cells was 79.19 for the entire cohort, 79.41 for the biphasic, 100.0 for the epithelioid, and 77.77 for the sarcomatoid subtype. Representative images of multispectral staining of pleural mesotheliomas are shown in Figure 2.

**Figure 3:**
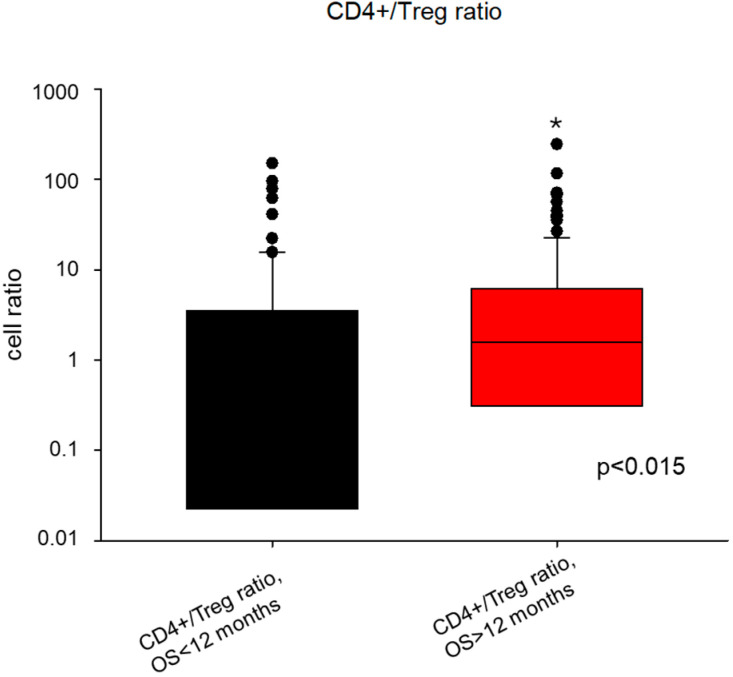
Comparison of the CD4+/Treg ratio of patients divided according to the 12-month overall survival cut-off. The median CD4+/Treg ratio was significantly higher in patients with an OS of more than 12 months than in those with a shorter OS (1.584 vs. 0.520; *p* = 0.015, indicated by *).

To evaluate the significance of the T cell immune infiltrate as a prognostic biomarker for pleural mesothelioma, the data were analyzed for associations with overall survival. A statistically significant difference between the CD4+/Treg ratio of patients with epithelioid and non-epithelioid histology was observed ([median (IQR)] 1.477 (0.255–6.547) versus 0.852 (0.000–2.850); *p* = 0.017). The median CD4+/Treg ratio was significantly higher in patients with an OS of more than 12 months than in those with a shorter OS (1.584 vs. 0.520; *p* = 0.015; Figure 3). In addition, the comparison of CD4+/CD8+ cell ratio showed a statistically significant difference between epithelioid and sarcomatoid subtypes (*p* = 0.028). 

The correlation of CD4+/CD8+ cell ratio with OS showed that patients with an OS of more than 18 months had a statistically significantly higher median CD4+/CD8+ cell ratio than patients with an OS of less than 18 months ([median (IQR)] 27.959 (21.935–40.533) versus 8.991 (5.654–12.977); *p* < 0.001). In the multivariate analysis, histological tumor subtype, gender, and age of patients had a significant impact on OS (*p* < 0.001, 0.050, and 0.006, respectively; Table 3). In addition, the ratio of CD4+ to Treg cells was still significantly associated with overall survival (*p* = 0.044). The ratio of CD4+/CD8+ cells continued to have a strong impact on OS (*p* = 0.059).

## 4. Discussion

In this study, we investigated the prognostic value of the T cell phenotypes present in the tumor-associated stroma of PM patients. The specific immune cells were analyzed using a multiplex immunochemical approach. We showed that the OS of patients who did not show PD-L1 expression in mesothelioma tissue was significantly better. Our initial results point to differences in the relevant immune cells in mesothelioma tissue that may influence tumor growth and disease progression. A high CD4+/Treg cell ratio was significantly associated with an OS of more than 12 months. In addition, a high ratio of CD4+/CD8+ cells was significantly associated with a longer survival time of more than 18 months. In the multivariate analysis, histologic subtype, gender, patient age and CD4+/Treg ratio had a strong impact on OS. As the introduction of immunotherapy has improved the treatment of non-epithelioid pleural mesothelioma, there is an urgent need for reliable biomarkers to predict disease progression and potential response to treatment in order to further optimize individualized treatment [2,13]. In addition, the epithelioid subtype, which responds very differently to immunotherapy in individual patients, could be better understood by analyzing the presence and influence of specific immune cell subsets [13]. Consequently, an analysis of the tumor microenvironment can help to identify relevant prognostic factors for disease progression and predict responses to therapy, allowing for a better stratification of therapeutic decisions. This can help to increase therapeutic efficacy and improve patient survival.

During the long latency period following exposure to asbestos, the interaction between immune cells and asbestos fibers results in pro-inflammatory processes with the predominant presence of tumor-associated macrophages (TAMs), which promote tumorigenesis and the inhibition of T cells [14,15]. Due to the shallow tumor growth along the parietal pleura, endothelial, stromal, and immune cells surround and invade the cancer cell area. It is possible that cancer cells recruit healthy cell types into their tumor microenvironment through specific mediators, so that these can serve as active collaborators in their malignant growth [16]. Tregs, a subset of CD4+ T cells, play an important role in maintaining immunological balance by suppressing various responses to self-antigens, infectious agents and tumors. The development and function of Tregs are controlled by the Foxp3 protein, which also serves as a specific diagnostic marker for their identification [17]. As previously described, the human pleural mesothelioma harbors a significant number of CD4+ T cells and Tregs [14]. Sarcomatoid tumors have lower levels of CD4+ and CD8+ T cells in the tumor tissue, which are required for an effective anti-tumor response [18]. Recent studies have shown that a high proportion of CD4+ T cells in epithelioid mesotheliomas is associated with a better prognosis. Conversely, a high expression of Tregs was associated with poor survival [19,20]. In our study, a higher CD4+/Treg ratio was associated with a longer OS. In view of the existing literature, this supports the hypothesis that a higher number of CD4+ T cells and a low number of Tregs seems to be a promising constellation for local tumor control. 

In contrast to NSCLC, patients with pleural mesothelioma have a better chance of survival if their tumors do not express PD-L1 [21,22]. Further analysis is still required to determine whether PD-L1 is a reliable marker of treatment response in patients receiving immunotherapy. Pathologic response rates in patients receiving immunotherapy followed by cytoreductive surgery for the treatment of pleural mesothelioma were analyzed in a recent study by Lee and colleagues. They identified the spatial immunologic architecture in mesothelioma tumor tissue as a determinant of significant pathologic response, which was associated with a PD-L1 expression greater than 5% [23]. In contrast, our results and the existing literature show that higher PD-L1 expression is observed in the sarcomatoid and biphasic subtypes and is negatively correlated with OS [18,22]. Whether starting immunotherapy early in the neoadjuvant phase can have an impact on tumor control in the different histological subtypes needs to be further investigated. Further research is also needed to determine how local and systemic T cell immunity interact in tumor control and how these two systems can be effectively manipulated to achieve effective tumor destruction.

One limitation of our study is that it is a retrospective cohort study. However, the use of this retrospective cohort allowed for a more homogeneous stratification of patients, as chemotherapy was the standard treatment, and the results were not influenced by immunotherapeutic treatment strategies. As grading of pleural mesothelioma has only been introduced into routine clinical practice in recent years, no information on this histopathologic feature was available for our study population. Consequently, a prospective analysis of mesothelioma patients may provide more information.

## 5. Conclusions

In the context of emerging immunotherapeutic strategies for the treatment of patients with pleural mesothelioma, the analysis of the local immune cell infiltrate is of increasing diagnostic importance. Detailed analysis of surgical tumor samples may lead to a better understanding of the underlying interactions of different immune cells that may have an impact on therapeutic mechanisms.

In the future, immune checkpoint inhibitors could be part of a multimodal treatment approach for selected patients. Treatment decisions could be based on a detailed analysis of the tumor microenvironment to stratify patients with regard to their potential response to treatment.

## Figures and Tables

**Table 1:** Multiplex immunofluorescence panel-based classification of various immune cells.

Cell Type	Multiplex Immunofluorescence Panel
CD3+ cells	DAPI+/CD3+
CD4+ cells	DAPI+/CD3+/CD4+
CD8+ cells	DAPI+/CD3+/CD8+
Regulatory T cells	DAPI+/CD3+/CD4+/CD8-/CD163-/FoxP3+
PD1 cells	DAPI+/PD1+
T helper cells	DAPI+/CD3+/CD4+/CD8-/CD163-/FoxP3-
DAPI cells	DAPI+

DAPI: 4′,6-Diamidin-2-phenylindol; PD: programmed cell death protein; CD: cluster of differentiation; FoxP3: Forkhead box Protein 3.

**Table 2:** Description of the study population.

Patient Characteristics		n (%)
Sex	Men	175 (93.1)
	Women	13 (6.9)
Age [years]	Median (IQR)	64.8 (59.0–72.0)
ECOG PS grade	0	52 (27.7)
	1	123 (65.4)
	2	13 (6.9)
Side	left	70 (37.2)
	right	118 (62.3)
Histologic tumor subtype	epithelioid	135 (71.8)
	sarcomatoid	9 (4.8)
	biphasic	44 (23.4)
Tumor stage (TNM8)	IB	153 (81.4)
	II	10 (5.3)
	IIIA	4 (2.1)
	IIIB	21 (11.2)
Exposure to asbestos	yes	123 (65.4)
	no	52 (27.7)
	unknown	13 (6.9)
PD-L1 expression	0%	91 (48.4%)
	1–49%	65 (34.6%)
	50–100%	32 (17.0%)
Survival status	Dead	188 (100)
Overall survival [months]	Median (IQR)	15.1 (8.3–25.4)

TNM: tumor, node, metastasis; IQR: interquartile range; ECOG PS: Eastern Cooperative Oncology Group Performance Status.

**Table 3:** Influence of different variables on survival (Cox regression analysis).

Variable	Hazard Ratio (95% CI)	*p*-Value
histology (ref. epithelioid)		
biphasic	2.046 (1.340–3.124)	<0.001
sarcomatoid	4.598 (1.983–10.664)	<0.001
age	1.926 (1.907–1.945)	0.006
ECOG PS (ref. 0)		
1	0.812 (0.566–1.166)	0.026
2	0.897 (0.482–1.668)	0.731
Gender (ref. female)		
male	0.543 (0.295–1.000)	0.050
CD4+/CD8+ ratio	0.854 (0.724–1.006)	0.059
CD4+/Treg ratio	1.207 (1.015–1.315)	0.044

ECOG PS: Eastern Cooperative Oncology Group Performance Status; CD: cluster of differentiation; CI: confidence interval.

## Data Availability

The original contributions presented in the study are included in the article; further inquiries can be directed to the corresponding author.
